# A case report of mental disorder caused by shunt blockage after hydrocephalus surgery

**DOI:** 10.3389/fpsyt.2024.1503993

**Published:** 2024-12-04

**Authors:** Jingjing Wu, Wei Li, Yaping Wang, Lijin Liu, Lanying Liu

**Affiliations:** ^1^ Department of Traditional Chinese Medicine, Shanghai Mental Health Center, Shanghai Jiao Tong University School of Medicine, Shanghai, China; ^2^ Xuzhou Oriental Hospital, Xuzhou Medical University, Xuzhou, China; ^3^ Department of Neurology and Psychology, Shenzhen Traditional Chinese Medicine Hospital, Shenzhen, China

**Keywords:** hydrocephalus, cerebral organic mental disorder, depression, NPH, VPS

## Abstract

**Introduction:**

Hydrocephalus is a form of communication hydrocephalus syndrome characterized by ventricular enlargement and normal intraventricular pressure. The primary clinical manifestations include gait disturbances, cognitive dysfunction, urinary incontinence, and either elevated or normal intracranial pressure. This paper presents a case of a mental disorder resulting from inadequate drainage following a ventriculoperitoneal shunt procedure for hydrocephalus. The case report aims to enhance clinicians’ understanding of such organic brain lesions, which are prone to misdiagnosis and inappropriate treatment, thereby improving differential diagnostic skills.

**Case presentation:**

This case report describes a 34-year-old male with a 16-year history of depressive disorder, previously managed with 150 mg of venlafaxine daily, 7.5 mg of zopiclone every night, and 2.4 g of piracetam every day. The patient underwent a ventriculoperitoneal shunt procedure for hydrocephalus, leading to the development of a mental disorder attributable to poor drainage from the shunt tube. Initial symptoms consisted of low mood, decreased interest, and cognitive impairment. Despite psychiatric consultation and antidepressant treatment, there was no improvement in his condition. The final diagnosis was an organic mental disorder. Following an increase in shunt drainage and the administration of a low dose of olanzapine, the patient’s psychiatric symptoms showed significant improvement.

**Conclusion:**

Reporting this case seeks to enhance clinicians’ awareness of the psychiatric manifestations of organic brain lesions, expand the differential diagnostic approach for psychiatrists, and improve diagnostic accuracy. Additionally, it emphasizes the need for cautious use of psychiatric medications, appropriate symptomatic management, and timely referral when necessary.

## Introduction

Hydrocephalus refers to the accumulation of cerebrospinal fluid within the ventricles and subarachnoid space, causing uneven ventricular expansion and brain dysfunction. This condition can manifest with symptoms such as headache, dizziness, vomiting, gait disturbances, altered consciousness, and urinary incontinence (1). Clinically, hydrocephalus is classified as either primary or secondary. Primary hydrocephalus is usually caused by abnormal cerebrospinal fluid circulation, while secondary hydrocephalus most commonly results from subarachnoid hemorrhage (2), followed by craniocerebral injury, intracranial tumors, and cerebrovascular diseases (3).

A meta-analysis has indicated that the mean prevalence of hydrocephalus is 85 per 100,000 individuals [95% CI, 62–116]. The prevalence is 88 per 100,000 [95% CI, 72–107] in pediatric populations, 11 per 100,000 [95% CI, 5–25] in adults, and 175 per 100,000 [95% CI, 67–458] in the elderly (4). Most patients with secondary hydrocephalus undergo ventriculoperitoneal shunt (VPS) surgery (5). However, postoperative complications, including infection and shunt blockage, are common, with a high incidence of shunt blockage leading to the recurrence of hydrocephalus symptoms (6).

When intracranial pressure remains within normal limits, the condition is termed normal pressure hydrocephalus (NPH), which is characterized by a triad of cognitive impairment, gait disturbances, and urinary incontinence (7). NPH is also typically treated with ventriculoperitoneal shunt (VPS). However, there are few research on the psychiatric symptoms associated with NPH. These symptoms always include apathy, anxiety, and depression, and they frequently present as the initial or primary manifestations, which can lead to diagnostic confusion with schizophrenia or depressive disorders (8).

This article describes a case of a patient who underwent a ventriculoperitoneal shunt procedure for hydrocephalus, resulting in a mental disorder due to poor drainage from the shunt. It is essential for clinicians to exercise caution in the use of psychiatric medications, provide appropriate symptomatic treatment, and consider timely referrals when necessary.

## Case presentation

The patient, a 34-year-old male, presented to the hospital with symptoms of low mood, decreased interest, poor sleep, and significant forgetfulness over the past two months, with a total history of 16 years of depressive symptoms. In 2003, he had a right subscalp shunt device placed. He also had a three-year history of hypothyroidism, for which he was not receiving treatment. Prior to 2008, the patient’s life and work were stable. However, in 2008, he began experiencing insomnia without an apparent cause, along with low mood, loss of interest, and minimal verbal communication. He was subsequently admitted to a local psychiatric hospital, where he was diagnosed with depressive disorder. After treatment with venlafaxine (150mg/day), zopiclone (7.5 mg/day) and piracetam (2.4 g/day), he was discharged after his condition improved and then discontinued regular medication for the next two years. During this period, he was able to work and perform daily tasks without significant issues. Since 2023, the patient reported a decline in work performance and learning ability, leading to frequent criticism from colleagues. In March 2024, he experienced increasing fatigue and resigned from his job following a conflict with colleagues. He reported persistent drowsiness, difficulty keeping his eyes open, forgetfulness, poor concentration, headaches, dizziness, and blurred vision. A craniocerebral MRI and blood tests performed at a local eye hospital in March 2024 did not reveal any abnormalities. However, due to continued daytime sleepiness, memory deterioration, irritability, and sleep disturbances, he was readmitted to a psychiatric hospital, where he was diagnosed with recurrent depressive disorder. Treatment was initiated with 150 mg of venlafaxine daily, 7.5 mg of zopiclone nightly, and 2.4 g of piracetam everyday.

Despite treatment, the patient’s condition did not improve, he remained unable to sleep at night, appeared agitated and disoriented, and had difficulty navigating the hospital with his eyes closed. His family perceived the treatment as ineffective, and he was discharged after five days. Following discharge, the patient continued to suffer from insomnia, excessive daytime sleepiness, inability to keep his eyes open, blurred vision, frequent urination, and significant memory impairment, to the point of being unable to recall his mobile phone payment password or differentiate time of day in the afternoon. These symptoms progressively worsened, prompting him to seek inpatient treatment at our hospital.

The patient denied any history of exposure to toxic or harmful substances. He was described as introverted and reported no family history of mental illness.

## Investigation

Admission Examination: cranial CT revealed an extension of the drainage tube in the right frontal region, approximately at the level of the lateral ventricles. The supratentorial ventricular system was notably widened, with blunt margins and altered tension. A symmetrical, low-density blurred area was observed surrounding the lateral ventricles. Additionally, a transparent septum was visible in the midline region. The bilateral lateral cisterns and sulci of the brain appeared significantly narrowed or absent, while the midline structures remained central.

**Figure 1:**
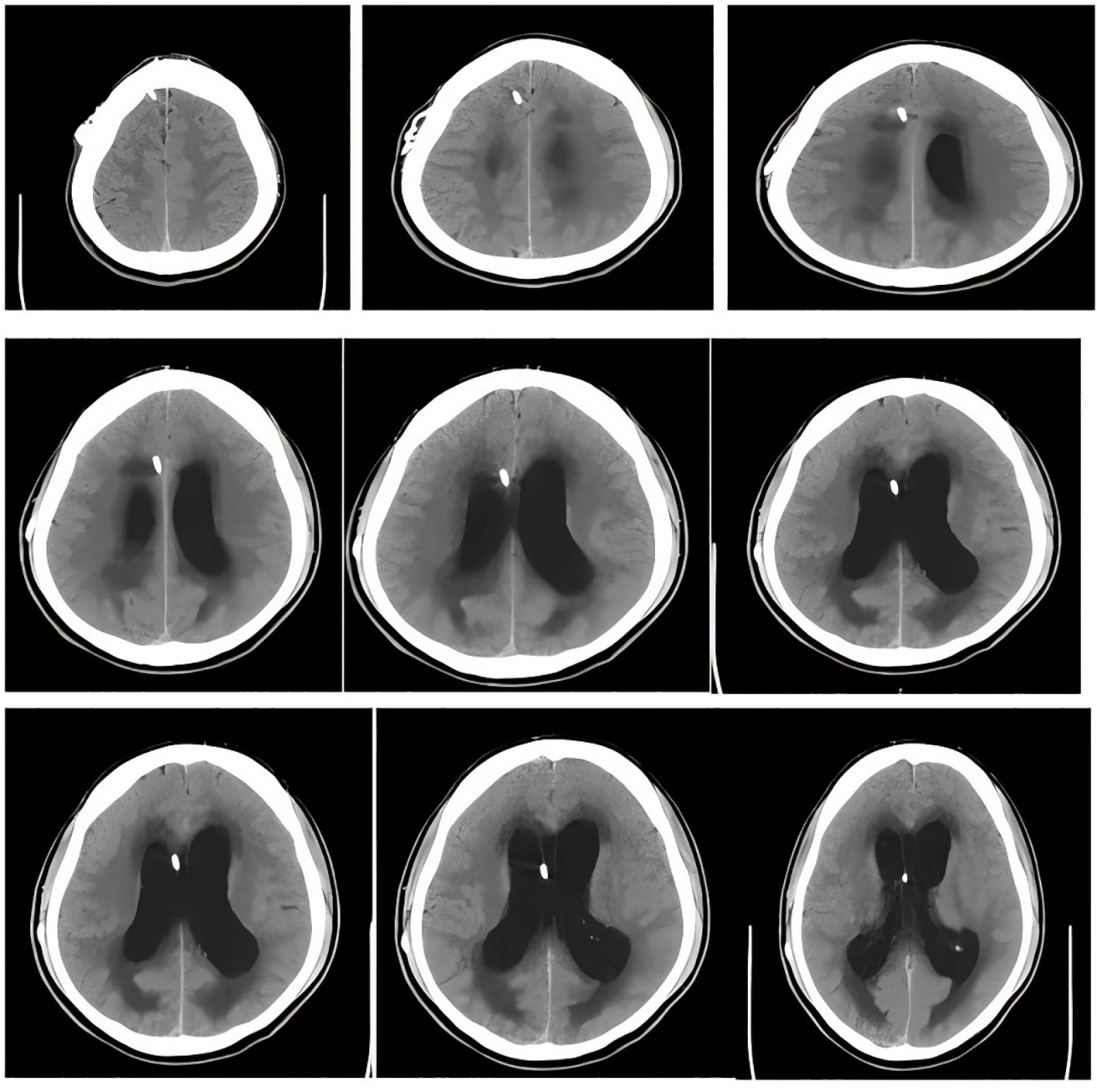
Head CT shows us the location of the shunt and significant widening of the ventricles and obvious symmetrical plaques in the lateral ventricles.

The radiological diagnosis was as follows: (1)supratentorial hydrocephalus, likely obstructive in nature; (2)brain swelling; (3)changes in the bilateral lateral ventricles. It is recommended to correlate these findings with clinical and MRI evaluations and to conduct close follow-up (refer to **Figure 1** for more details).

Mental examination on admission: The patient was alert and oriented, but with impaired time and place orientation. While he could engage in conversation, his concentration was diminished, leading to more questions being asked than answered. His tone of voice was low, and there was no evidence of hallucinations, illusions, or other sensory perception disturbances. He reported various physical discomforts, including dizziness and headache, along with blurred vision. There was noticeable psychomotor retardation, but no apparent disturbances in logical thinking. He exhibited symptoms suggestive of delusional thinking, including ideas of possession. The patient showed signs of anhedonia, fatigue, loss of energy, anxiety, and restlessness, though his emotional responses remained appropriate. There was evidence of social withdrawal and cognitive decline, with preserved long-term memory but significant impairments in recent and immediate memory. He also demonstrated confabulation and disorganized thinking, reduced computational ability, and limited insight into his condition.

Physical examination: Cardiopulmonary auscultation was unremarkable. Neurological examination revealed clear consciousness, though the patient appeared indifferent and responded to questions slowly. He cooperated with the examination despite reduced orientation to time and space, while memory and calculation abilities remained intact. Both eyes showed normal vision without visual field defects, and there was no abnormality in the fundus. The eyelids were not drooping, and ocular movements were normal. The pupils were bilaterally symmetrical, approximately 3 mm in diameter, and reactive to both direct and indirect light, as well as accommodation. There was no evidence of atrophy or abnormal movements during mastication, and the corneal reflex was intact.

The facial muscles, including the frontal lines, frown, and nasolabial folds, appeared normal, with strong bilateral eyelid closure. Hearing was intact bilaterally, and the soft palate was symmetrical with a normal posterior pharyngeal wall and present pharyngeal reflex. The patient exhibited strong bilateral neck rotation and shoulder shrug, and his tongue extended to the midline without tremor or atrophy. There were no signs of muscle atrophy or pseudohypertrophy in the limbs, and muscle strength was normal across all extremities, without tremors. The patient could move all limbs autonomously, although his gait was unsteady. Coordination tests, including the finger-to-nose test, knee-heel test, and rapid alternating movements, were normal, with a positive Romberg test indicating postural instability. Pain perception, vibration sensation, and proprioception were intact bilaterally, and compound sensory functions were normal. Tendon reflexes in all four limbs were normal, and the abdominal wall reflex was intact. Bilateral Babinski, Chaddock, Oppenheim, Gordon, and Hoffman reflexes were positive. There was no evidence of neck stiffness, and both Kernig’s and Brudzinski’s signs were negative. The patient’s skin showed normal color and temperature, sweat gland activity was appropriate, hair distribution was uniform, and the skin scratch test was normal. Several scales were evaluated, including Hamilton Depression Rating Scale (HAMD-17) (score18, moderate depressive symptoms), Negative Syndrome Scale (PANSS) (49 points, showing low mood, paying attention to physical health, social withdrawal) (**Table 1**).

**Table 1:** Changes in dosage of venlafaxine (mg), olanzapine(mg), Piracetam (g), Positive And Negative Syndrome Scale(PANSS), Hamilton Depression Rating Scale (HAMD-17).

Date	31/3	1/4	2/4	3/4	15/4
venlafaxine(mg)	150	150	0	0	0
olanzapine (mg)	0	2.5	2.5	2.5	0
Piraceta (g)	2.4	2.4	2.4	2.4	2.4
PANSS score		49			37
HAMD-17score		18			12

## Diagnosis

Following admission, and considering the patient’s medical history, clinical presentation, examination findings, and physical assessment, the diagnosis included the classic triad of normal pressure hydrocephalus (NPH): cognitive impairment, gait disturbance, and urinary incontinence. Additionally, the recent onset of memory deficits, confabulation, and disorientation was consistent with features of chronic encephalopathy syndrome (Korsakoff syndrome). The presence of psychiatric symptoms, including significant nighttime irritability and delusions, indicated an acute encephalopathy syndrome. Based on the ICD-10 diagnostic criteria, the final diagnoses were cerebral organic mental disorder, normal pressure hydrocephalus, and status post ventriculoperitoneal shunt.

## Therapeutic intervention

The use of antidepressants was discontinued, and the patient was started on 2.5 mg of the antipsychotic olanzapine daily to enhance cognitive function, stabilize mood, and improve sleep. Following this treatment, there was partial improvement in the patient’s sleep and mood, along with reduced irritability. However, the patient continued to experience frequent urination, blurred vision, and gait instability. A head CT revealed significant abnormalities, and the neurological examination indicated pathological findings suggestive of a possible blockage in the drainage tube.

Given these concerns, the patient’s family arranged for a transfer to the neurology department of another hospital for further evaluation, leading to his discharge. On the day of discharge, the patient presented to the emergency department of a general hospital in Shanghai, where examination confirmed a blockage in the ventriculoperitoneal shunt. The patient was treated with 250 ml of mannitol for hydrocephalus and underwent catheterization to relieve urinary retention.

### Outcomes and follow-up

Following the infusion, the patient’s condition improved; he was able to open his eyes and follow instructions. The family was advised to increase the frequency of shunt drainage, which led to gradual symptom improvement. Based on the follow-up and evaluation on April 15^th^, the patient regained the ability to urinate normally, walk with stability, experienced significantly reduced drowsiness, and maintained a stable mood. He was able to perform simple household tasks, although slight dizziness persisted. Consequently, the olanzapine was discontinued, and the patient continued on 2.4 g of piracetam daily to address residual cognitive dysfunction (**Table 1**). The patient and their family are very grateful for our correct diagnosis and medication.

## Discussion

Review of the Diagnosis and Treatment Process: The patient underwent a ventriculoperitoneal shunt procedure for hydrocephalus during childhood. However, there was a long period during which he did not receive follow-up care in the neurology department. The shunt device was implanted subcutaneously under the scalp, making it difficult to detect externally. Both the patient and his family believed he had fully recovered, and thus, his medical history was not thoroughly reviewed during subsequent consultations. Consequently, the initial symptoms of hydrocephalus recurrence did not present as the typical triad associated with normal pressure hydrocephalus (NPH).

In 2008, the patient was hospitalized with mental health symptoms, including persistent depression and poor sleep, which were initially diagnosed as depressive disorder. However, further consultation and detailed examination revealed that the onset of symptoms lacked a clear precipitating cause. By March 2024, the patient had been diagnosed with recurrent depressive disorder at a local hospital. Following treatment with antidepressants, his condition worsened, with increased irritability and fatigue, leading to a decline in work performance. The presence of both cognitive impairment and mood-related symptoms warranted consideration of an alternative diagnosis.

Currently, there is substantial research on gait, cognitive, and urinary dysfunction in patients with NPH (9). Neuropsychiatric symptoms have emerged as a key aspect of NPH pathology. Studies have shown that these symptoms primarily involve frontal lobe dysfunction (10), resulting in diminished cognitive abilities such as attention, apathy, changes in executive function, and personality traits that resemble those seen in Alzheimer’s disease (11). Furthermore, NPH patients often experience apathy, anxiety, and depression, which may be linked to underlying cognitive impairment (12). A Swedish study on the risk factors and complications of NPH found a 46% prevalence of depressive symptoms among patients who had undergone shunt surgery (13). These affective symptoms, along with the three primary clinical manifestations, generally improve following shunt surgery (14).

Hideki et al. (15) confirmed that apathy in NPH patients correlates with dysfunction in the frontal-subcortical circuitry, particularly involving the right caudate nucleus, suggesting that the same neural network may underlie both cognitive and psychiatric symptoms (8). In Japan, apathy is recognized as the fourth cardinal symptom of NPH, highlighting the importance of accurate clinical identification (16). As a result, indifference, anxiety, and depression are frequently encountered in NPH patients. It is crucial for psychiatrists to differentiate these symptoms appropriately to avoid misdiagnosis, treatment delays, or incorrect medication, which could worsen the condition.

The standard surgical intervention for hydrocephalus is the ventriculoperitoneal shunt (17), with postoperative complication rates ranging from 24% to 52% (18). Shunt failure is among the most common complications, with a 10-year incidence exceeding 60% (19). Shunt blockage remains the predominant cause of hydrocephalus recurrence, leading to symptoms such as dizziness, headache, gait instability, and altered consciousness; less commonly, patients may present with mutism, dystonia, or bradykinesia (20). Neurological examinations in such cases often reveal abnormalities, while imaging studies typically show ventricles enlargement and signs of compressed brain parenchyma.

The clinical diagnosis of shunt blockage is based on the recurrence of hydrocephalus symptoms that were present before surgery. Diagnostic criteria include: 1) difficulty filling the valve or puncturing it, indicating a blocked shunt; 2) lack of smooth flow through the shunt; 3) CT or MRI evidence of effusion around the shunt, with unchanged or expanded ventricles, and blunt angles at the ventricular borders; and 4) the appearance of fluid when compressing the shunt pump. When blockage occurs, the ratio of cortical thickness to lateral ventricular width may reach 1:1. The presence of two or more of these findings suggests a shunt obstruction (21).

If the progression of organic mental disorders is not promptly and effectively managed, severe cognitive decline can ensue, potentially leading to life-threatening conditions or dementia (22). Among the pharmacological options for treating brain organic mental disorders, atypical antipsychotics such as olanzapine and risperidone have demonstrated favorable efficacy. Olanzapine, a derivative of thiophene benzodiazepine, has a high affinity for several receptors, including dopamine (DA) and histamine H1 receptors, allowing it to selectively modulate the DA pathway in the brain’s limbic regions. This mechanism significantly alleviates psychiatric symptoms (23), with fewer adverse effects in comparison to other treatments. Therefore, low-dose olanzapine is considered effective in managing mental disorders resulting from organic brain disease (24). However, regarding the clinical use of olanzapine, in 2005, based on a meta-analysis of randomized controlled trial (RCT) data, the FDA issued a warning that second-generation antipsychotic treatment of the behavioral disturbances associated with dementia was associated with increased mortality (25). Therefore, in the future, a more cautious attitude should also be taken when selecting drugs for elderly patients with mental disorders.

## Limitations

Due to the fact that this study is a non RCT observational case report, the lack of comparison between control group and post-treatment brain imaging reports has limited the persuasiveness of the research results, and more series of case reports are needed in the future to provide evidence. Meanwhile, due to the lack of involvement of clinical pharmacists in clinical decision-making, drug-related psychiatric symptoms have occurred (26, 27). Therefore, in the future, more recommendations from clinical pharmacists need to be adopted to guide clinical medication. In addition, due to the lack of relevant cognitive assessment after the occurrence of cognitive impairment in patients, there is a lack of specific dimensions of cognitive impairment in patients with normal intracranial pressure hydrocephalus.

## Conclusion

In summary, psychiatric symptoms are a significant aspect of the clinical presentation of organic brain lesions and may manifest before abnormalities are detectable through imaging. By presenting this case, we aim to enhance clinicians’ awareness of the psychiatric manifestations associated with organic brain lesions, expand the differential diagnostic framework for psychiatrists, and improve diagnostic accuracy. It is essential for clinicians to exercise caution in the use of psychiatric medications, provide appropriate symptomatic treatment, and consider timely referrals when necessary.

## Data Availability

The original contributions presented in the study are included in the article/supplementary material. Further inquiries can be directed to the corresponding authors.
